# Therapeutic Potential of Dimethyl Fumarate for the Treatment of High-Fat/High-Sucrose Diet-Induced Obesity

**DOI:** 10.3390/antiox13121496

**Published:** 2024-12-08

**Authors:** Helber da Maia Valenca, Evelyn Caribé Mota, Andressa Caetano da Fonseca Andrade Silva, Alexsandro Tavares Figueiredo-Junior, Fernanda Verdini, Bruna Romana-Souza, Mariana Renovato-Martins, Manuella Lanzetti, Samuel dos Santos Valenca, João Alfredo Moraes

**Affiliations:** 1 Institute of Biomedical Sciences, Federal University of Rio de Janeiro (UFRJ), Avenida Carlos Chagas Filho 373, bloco F, 3° floor, room 301, Cidade Universitária, Rio de Janeiro CEP 21941-902, RJ, Brazil; helfarma@yahoo.com.br (H.d.M.V.); evelyncaribe5@gmail.com (E.C.M.); dessa1205@gmail.com (A.C.d.F.A.S.); figueiredojunior.at@gmail.com (A.T.F.-J.); nandaverdini22@yahoo.com.br (F.V.); manuellalanzetti@icb.ufrj.br (M.L.); joaomoraes@icb.ufrj.br (J.A.M.); 2 Department of Histology and Embryology, State University of Rio de Janeiro (UERJ), Rua Professor Manoel de Abreu, 444, 3° andar, Rio de Janeiro CEP 20550-170, RJ, Brazil; romanabio@gmail.com; 3 Laboratory of Inflammation and Metabolism, Biology Institute, Departament of Cellular and Molecular Biology, Fluminense Federal University (UFF), Rua Professor Marcos Waldemar de Freitas Reis, s/n, Campus do Gragoatá, Bloco M, room 316, Niterói CEP 24210-201, RJ, Brazil; marianarenovato@id.uff.br

**Keywords:** obesity, dimethyl fumarate, monomethyl fumarate, high-fat and high-sucrose diet, oxidative stress

## Abstract

Obesity is characterized by an imbalance between energy intake and expenditure that triggers abnormal growth of adipose tissues. Dimethyl fumarate (DMF) and its primary active metabolite, monomethyl fumarate (MMF), are Nrf2 activators and have been recognized as strategic antioxidants. This study aimed to evaluate the potential of MMF and DMF to interfere with adipogenesis and obesity, and identify the molecular mechanisms involved. The 3T3-L1 preadipocytes were incubated with differentiation medium (MIX) and simultaneously treated with different concentrations of MMF. In addition, male C57BL/6 mice were fed a standard diet or high-fat/high-sucrose diet (HFHSD) for 16 weeks, during the last 4 of which, they received oral DMF treatment. Exposure to MMF prevented the development of MIX-induced adipogenesis by reducing the expression of transcription factors that drive adipocyte differentiation and by decreasing triglyceride levels. In addition, various antioxidant and anti-inflammatory effects were observed after treatment with MMF as evidenced by the modulation of transcription factor activities and reduction in reactive oxygen species, adipokine, proinflammatory cytokine and resistin levels. In vivo treatment with DMF reduced calorie intake, body weight, and visceral and subcutaneous fat mass in HFHSD mice. Furthermore, DMF administration led to a better glycemic response as well as lower leptin and adiponectin plasma levels in these animals. Our data demonstrate that DMF and its metabolite MMF interfere with adipogenesis and prevent the key features of diet-induced obesity. Considering DMF is already a commercial drug used to treat psoriasis and multiple sclerosis, its pharmacological application for the treatment of obesity and related metabolic disorders holds promise.

## 1. Introduction

Obesity is a complex and multifactorial medical condition characterized by an excessive accumulation of adipose tissue in the body, resulting in a significant increase in body weight [1]. It has become a major global public health concern because of its increasing prevalence and significant implications for individual as well as collective health [2]. Obesity is strongly linked to a series of health complications, including cardiovascular disease, type 2 diabetes, hypertension, metabolic disorders, and certain types of cancer, which are among the ten leading causes of death worldwide [3]. Furthermore, obesity has considerable socioeconomical effects, impacting quality of life, healthcare costs, and productivity [4].

In addition to an excessive accumulation of adipose tissue, low-grade inflammation has also been recognized as a typical feature of obesity. Increased metabolic activity in response to excess nutrients such as fatty acids leads to the production of free radicals, including superoxide anions and hydrogen peroxide, by adipocytes [5]. Furthermore, the inflammation present in adipose tissue also contributes to the production of reactive oxygen species (ROS) through the activation of enzymes like nicotinamide adenine dinucleotide phosphate hydrogen (NADPH) oxidase [6]. ROS can activate inflammatory signaling pathways (e.g., nuclear factor kappa B [NF-κB]) and promote the release of proinflammatory cytokines. Indeed, oxidative stress induces adipose tissue dysfunction resulting in exacerbated release of proinflammatory adipokines and reduced adiponectin secretion, thereby contributing to local and systemic inflammation. In addition, oxidative stress impacts the lipid metabolism by promoting lipogenesis and inhibiting lipolysis; these metabolic changes are associated with ectopic fat accumulation and insulin resistance [7].

Chronic inflammation associated with obesity plays a key role in the pathogenesis of many metabolic complications observed in obese individuals [8]. Proinflammatory macrophages are particularly abundant, contributing to the release of cytokines and amplifying the local inflammatory response [9]. Furthermore, adipokines secreted by adipose tissue, such as adiponectin and leptin, also have inflammatory and metabolic functions. Adiponectin, with anti-inflammatory and insulin-sensitizing properties, is reduced in obese individuals, thus favoring inflammation [10]. In addition, leptin, which regulates appetite, contributes to inflammation when secreted in excess [11]. Chronic inflammation is also associated with insulin resistance and the development of type 2 diabetes [12]. An increase in proinflammatory cytokines impairs insulin signaling, compromising cellular glucose uptake and leading to hyperglycemia [13]. Because chronically inflamed adipose tissues typically release free fatty acids and proinflammatory adipokines that contribute to liver inflammation, necrosis, and fibrosis, nonalcoholic fatty liver disease (NAFLD) and nonalcoholic steatohepatitis (NASH) are comorbidities frequently associated with obesity [14].

Dimethyl fumarate (DMF) is an ester synthesized from fumaric acid that is used as an acidulant and nutritional additive in the food and feed sector [15]. The esterification process of fumaric acid occurs through its reaction with two methanol molecules, rendering DMF and two water molecules. DMF has gained significant interest in the medical field because of its potential therapeutic properties. For example, it is known for its anti-inflammatory and antioxidant actions and has been used to counteract pathological conditions involving oxidative stress and inflammation [16]. Mechanistically, DMF acts as an activator of the nuclear factor erythroid 2–related factor 2/antioxidant response element (Nrf2/ARE) signaling pathway, playing a fundamental role in the cellular antioxidant response. DMF covalently binds to the reactive cysteine residues of kelch-like ECH-associated protein 1 (Keap1) through a Michael reaction, preventing the continuous degradation of newly synthesized Nrf2. This allows Nrf2 to accumulate in the cytoplasm and subsequently translocate to the nucleus, where it regulates the expression of cytoprotective genes [17]. DMF is currently used as the active ingredient in two medications for the treatment of psoriasis (Fumaderm^®^) and multiple sclerosis (Tecfidera^®^) [18]. When administered orally, DMF undergoes rapid pre-systemic hydrolysis by intestinal carboxylesterases and is converted into an active metabolite, monomethyl fumarate (MMF) [19]. The wide use of DMF, however, should be accompanied by significant safety concerns, including the potential risk of progressive multifocal leukoencephalopathy [20]. While DMF has shown promising therapeutic effects in preclinical models, safety issues underscore the need for caution when translating these findings into clinical applications [21]. Our study focuses on the preclinical evaluation of DMF’s effects, which provides valuable insights into its mechanisms of action and therapeutic potential [22]. However, we acknowledge that the findings do not directly translate to clinical applicability without addressing these significant safety concerns [23]. We aim to ensure transparency and avoid creating unrealistic expectations for patients or clinicians. Here, we hypothesized that DMF can regulate metabolism in mice subjected to a high fat/high sucrose diet (HFHSD).

## 2. Materials and Methods

### 2.1. Culture and Differentiation of 3T3-L1 Cells

**Figure 1:**
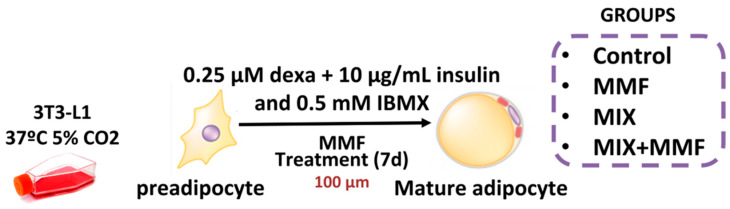
Schematic of the in vitro model of adipocyte maturation. The 3T3 cells were cultured in Dulbecco’s modified eagle medium (DMEM) supplemented with 10% calf serum (CS) and divided into separate groups. To induce adipocyte maturation, cells were kept in differentiation mixture (MIX) for 7 days. Control and MIX groups were cultured in the absence and presence of various concentrations of monomethyl fumarate (MMF) to inhibit adipogenesis.

All reagents used in this study were purchased from Sigma-Merck (São Paulo, Brazil) unless indicated otherwise. Murine 3T3-L1 preadipocytes were cultured in Dulbecco’s modified eagle medium (DMEM) supplemented with 10% calf serum (CS); differentiation was subsequently induced by culturing cells in medium supplemented with a differentiation mixture composed of insulin (10 µg/mL), dexamethasone (0.25 µM), and 3-Isobutyl-1-methylxanthine (IBMX) (0.5 mM) for 7 days. To determine the effect of MMF on adipogenesis, groups of cells were incubated with 10, 30, or 100 µM MMF, after which the accumulation of intracellular lipids was evaluated via Oil Red O staining. A concentration of 100 µM MMF was subsequently selected for further in vitro tests, as it was shown to be the most effective. Preadipocytes were divided into the following experimental groups: control—cells cultured in DMEM; control + MMF—cells cultured in DMEM + MMF (100 µM); MIX—cells cultured in differentiation mixture; and MIX + MMF group. The study design is illustrated in Figure 1.

### 2.2. Oil Red O Staining

The accumulation of intracellular lipids was detected via Oil Red O staining. After the seventh day of adipogenesis, medium was removed, and the adipocytes were washed twice with phosphate-buffered saline (PBS) and fixed with 10% formalin for 30 min at room temperature. Next, formalin was aspirated and an Oil Red O working solution 3% (*w*/*v*) in 60% (*v*/*v*) isopropanol in water was added; plates were incubated for 20 min at 37 °C and then aspirated. Subsequently, adipocytes were washed twice again with PBS and 99% commercial isopropyl alcohol was added to the wells. The absorbance of the supernatant was measured at 520 nm using a plate reader (FlexStation 3 Multi-Mode Microplate Reader, Molecular Devices Inc., San Jose, CA, USA).

### 2.3. 3-(4,5-Dimethylthiazol-2-yl)-2,5-Diphenyl Tetrazolium Bromide (MTT) Test

Preadipocyte viability was assessed via MTT assays at various time points during adipogenesis (24, 48, and 96 h). Cells were seeded in 96-well plates at a density of 1 × 10^4^ cells per well and exposed to different concentrations of MMF (10, 30, and 100 µM) in DMEM supplemented with 1% CS. At each time point (24, 48, and 96 h), cells were incubated in the dark with MTT solution (5 mg/mL) for 4 h at 37 °C. Next, supernatants were removed, isopropyl alcohol was added to each well, and the plate was agitated to dissolve the formazan crystals. Absorbance was read at 570 nm using a plate reader. The percentage of viable cells was determined by setting the viability of untreated (DMEM only) cells to 100%.

### 2.4. Determination of Triglyceride Content

Triglyceride levels in mature adipocyte extracts were measured using a colorimetric enzymatic assay kit (Bioliquid^®^ kit [#743191], Laborclin Produtos para Laboratórios LTDA, Pinhais, Brazil) according to the manufacturer’s protocol.

### 2.5. ROS Assay

Preadipocytes were plated in 96-well plates at a density of 1 × 10^4^ cells per well for 24 h and treated with different concentrations of MMF (10, 30, and 100 µM) in Hank’s balanced salt solution (HBSS) medium containing a specific probe for ROS detection (2′,7′-dichlorodihydrofluorescein diacetate [H2DCFDA]—10 μM). Lipopolysaccharide (LPS) was used as a positive control for ROS production (1 µg/mL for 24 h). After incubation at 37 °C and 5% CO_2_ for 1 h (to allow the probes to internalize), supernatants were removed, and cells were washed to remove non-internalized probes. Next, cells were treated as described above and immediately placed in a black 96-well plate for stimulation and monitoring of probe oxidation using a Varioskan LUX (Thermo Fisher Scientific Inc., Waltham, MA, USA) plate reader at excitation and emission fluorescent wavelengths of 495 and 525 nm, respectively.

### 2.6. Cytokine Quantification

Adipocyte supernatants were analyzed for interleukin (IL)-6 levels (#900-T50K). In addition, plasma samples were used to determine the concentrations of IL-6, IL-1β (#900-K47K), tumor necrosis factor (TNF)-α (#900-K54K), adiponectin (#900-K74K), leptin (#900-K76K) and glutamate pyruvate alanine aminotransferase (GPT) (#900-K78K). These analyses were performed using enzyme-linked immunosorbent assay (ELISA) kits following the manufacturer’s instructions (PeproTech Inc., Ribeirão Preto, Brazil).

### 2.7. Luciferase Assay for the NF-κB and Nrf2/ARE Pathways

The 3T3-L1 cells were seeded in 24-well plates in DMEM/F12 containing 10% CS. The next day, cells were transfected with a luciferase reporter responsive to NF-κB (NF-κB reporter kit [#60614]) or Nrf2/ARE (BPS Bioscience, San Diego, CA, USA) in Opti-MEM culture medium (Gibco, Billings, MT, USA) containing Lipofectamine 2000 (Thermo Fisher Scientific Inc., Waltham, MA, USA) and incubated for 24 h. After stimulation with MIX, LPS and MMF, the cells were incubated for another 24 h, after which the medium containing luciferase was collected. The mixture was then incubated with luciferin-specific substrates (Dual-Luciferase Assay System) (BPS Bioscience, San Diego, CA, USA) according to the manufacturer’s instructions. Luminescence emitted by luciferin cleavage was measured using a Varioskan LUX plate reader (Thermo Fisher Scientific Inc., Waltham, MA, USA).

### 2.8. Animals and Diet

After approval from the ethics committee (CEUA–137/19), 34 male C57BL/6 mice, aged 8 weeks (23–26 g), were purchased from the Multidisciplinary Center for Biological Research in the Area of Science in Laboratory Animals (CEMIB/UNICAMP, Campinas, Brazil). Animals were placed in groups of 7–10 per cage, with controlled temperature and humidity (21 ± 2 °C and 50 ± 10%, respectively), and subjected to 12 h light/dark cycles (artificial lights, 7 a.m.–7 p.m.).

The C57BL/6 mouse strain is susceptible to HFHSD-induced obesity and type 2 diabetes [24]. In addition, we used male mice because they tend to develop obesity and related metabolic disorders more consistently when fed a high-fat diet. Their hormonal profile is more stable compared to females, making them less prone to fluctuations caused by estrous cycles, which can introduce variability in metabolic studies [25].

The high-fat/high-sucrose (HFHSD) and standard diets were prepared by PRAGSOLUÇÕES^®^ (São Paulo, Brazil) in accordance with the recommendations of the American Institute of Nutrition (AIN-93 M) and offered to the animals ad libitum. The dietary compositions were: 20% protein, 72% carbohydrate, and 8% lipid (standard diet); 20% protein, 35% carbohydrates, and 45% lipids (HFHSD). The standard diet provided 38 kcal for every 10 g consumed, while the HFHSD diet provided 47 kcal for every 10 g consumed. For detailed composition of HFHSD see the Supplementary Materials (Figures S1 and S2).

### 2.9. In Vivo Experimental Design

**Figure 2:**
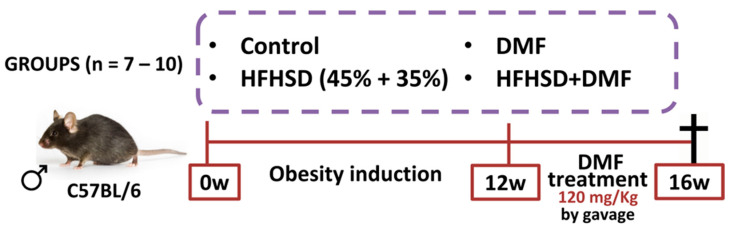
In vivo protocol of obesity induction and dimethyl fumarate (DMF) treatment. Obesity was induced by feeding mice a high-fat/high-sucrose diet (HFHSD) diet over 12 weeks. Treatment with DMF, given by gavage once daily, was initiated at the start of week 13 and continued until the end of week 16. All mice were euthanized at the end of the protocol to collect biological material for further analyses.

Animals were divided into separate groups: control (fed a standard diet); control + DMF (standard diet and DMF treatment); HFHSD (fed HFHSD); and HFHSD + DMF (fed HFHSD and DMF treatment). The in vivo protocols lasted for 16 weeks, with 12 weeks for obesity induction and 4 weeks of treatment with DMF (Figure 2). DMF was suspended in methylcellulose (0.8%) and given by oral gavage at a dose of 120 mg/kg once daily, based on a previous study with slight modifications [26]. All other mice not treated with DMF received vehicle (methylcellulose). Body weight was measured daily for 16 weeks. After euthanasia, the blood, left tibia, subcutaneous and visceral adipose tissues, and the liver were removed from each mouse for histology, biochemical and biomolecular analyses. Food and liquid intake were measured daily for 16 weeks by subtracting remaining food and drink provided from the mouse cage. At the end of the experiment, mice received an overdose of ketamine and xylazine to ensure the animal was deeply anesthetized and insensible to pain followed by exsanguination by cardiac puncture.

### 2.10. Glucose Monitoring, Glucose Tolerance Testing, and Insulin Resistance Testing

Glucose levels were monitored every two weeks from the start of DMF treatment. The mice were fasted for a period of 6 h. Next, blood samples were collected from the tail and glucose was measured using an Accu-Chek^®^ (Roche Diabetes, São Paulo, Brazil) digital device. The glucose tolerance test was performed 3 days before euthanasia. After fasting for 6 h, a 50% glucose solution was administered intraperitoneally at a dose of 1 g/kg body weight. Blood samples were collected from the tail and analyzed at 0, 15, 30, 60, 90, and 120 min after glucose injection. Insulin resistance test was performed one day before euthanasia. Insulin (Humulin^®^ R, Eli Lilly do Brasil Ltda., São Paulo, Brazil) was administered intraperitoneally at a dose of 0.54 IU/kg of body weight. Blood samples were collected from the tail and analyzed at 0, 15, 30, 60, 90, and 120 min after insulin injection.

### 2.11. Hematoxylin and Eosin (H&E) Staining

The sections (live and fat tissues) were deparaffinized in two xylene baths, hydrated in a series of decreasing concentrations of ethyl alcohol (100%, 90%, and 70%), and washed in distilled water. Staining was carried out with Harris hematoxylin for 10 s, followed by washing in running water for 5 min. Sections were subsequently stained with eosin for 10 s, quickly washed in distilled water, dehydrated in ethyl alcohol (70%, 90%, and 100% alcohol), clarified in two xylene baths, and mounted on coverslips with Entellan^®^ (Merck Millipore Brasil, Barueri, Brazil). The slides were analyzed under a light microscope with a 40× objective; 10 fields were captured per slide.

### 2.12. Histological Analysis of Visceral and Hepatic Adipose Tissues

Slides stained with H&E were analyzed under a light microscope with a 40× objective, capturing 10 fields per slide of visceral and hepatic adipose tissue. The morphological profile of adipocytes (hyperplasia or hypertrophy) was evaluated by assessing the average cell diameter (five adipocytes per field) via ZEISS Axiolab 5 (Carl Zeiss Microscopy GmbH, Jena, Germany). The quantification of lipids in liver tissue was conducted via the M42 test system, which comprises a transparent grid with 42 test points projected to a monitor [27]. To assess the degree of hepatic steatosis, the points intersecting with the lipids in the 10 fields of the slide were counted.

### 2.13. Electrophoresis and Western Blotting

Cellular lysates (10–20 μg of protein) were fractionated by denaturing 10–12% sodium dodecyl sulfate-polyacrylamide gel electrophoresis (SDS‒PAGE) and separated based on different molecular weights. Proteins were transferred (25 V, 1.0 A, and 30 min) to a polyvinylidene difluoride (PVDF) membrane via transfer buffer containing methanol using Trans-Blot Turbo^TM^ equipment (Bio-Rad Laboratórios Brasil Ltda., São Paulo, Brazil). The membranes were subsequently blocked with 5% bovine serum albumin (BSA) in 0.1% tris-buffered saline with tween 20 (T-TBS) for 1 h and incubated overnight at 4 °C with primary antibodies (please see Table 1 for antibodies and dilutions). After thorough washing, the membranes were exposed to a specific secondary antibody conjugated to peroxidase for at least 1 h. Immunoreactive proteins were visualized using enhanced chemiluminescence (ECL) solution. The membranes were developed and photographed using an ImageQuant^TM^ LAS500 (GE Healthcare Bio-Sciences Corp., Piscataway, NJ, USA), and densitometry was quantified by ImageJ software (version 1.54j 12 June 2024, developed at the National Institutes of Health). All bands were normalized to β-actin expression. Uncropped membranes are shown in the Supplementary Materials (Figure S3).

### 2.14. Statistical Analysis

The results were calculated as the mean ± standard deviation (SD) and analyzed via analysis of variance (ANOVA) followed by a Bonferroni post hoc correction, when appropriate. Statistical analyses were performed using GraphPad Prism (GraphPad Software Version 9.0.0, Boston, MA, USA). Differences were statistically significant when *p* < 0.05.

## 3. Results

### 3.1. MMF Does Not Affect Cell Viability at Any Concentration or Duration of Incubation

MTT assay results revealed that MMF did not affect the viability of 3T3-L1 cells at concentrations of 10, 30, and 100 µM after 24, 48, and 96 h (Figure S4). The results were statistically like those of the control group. DMSO was used as the solvent for diluting MMF and did not impact the metabolic viability of the cells at any of the concentrations tested. There were no significant differences observed between the experimental groups. Compared with the control group (DMEM 1%), the DMEM 10% CS group (positive control) presented higher proliferation rates.

### 3.2. MMF Prevents Adipogenesis at Concentrations of 30 and 100 µM

**Figure 3:**
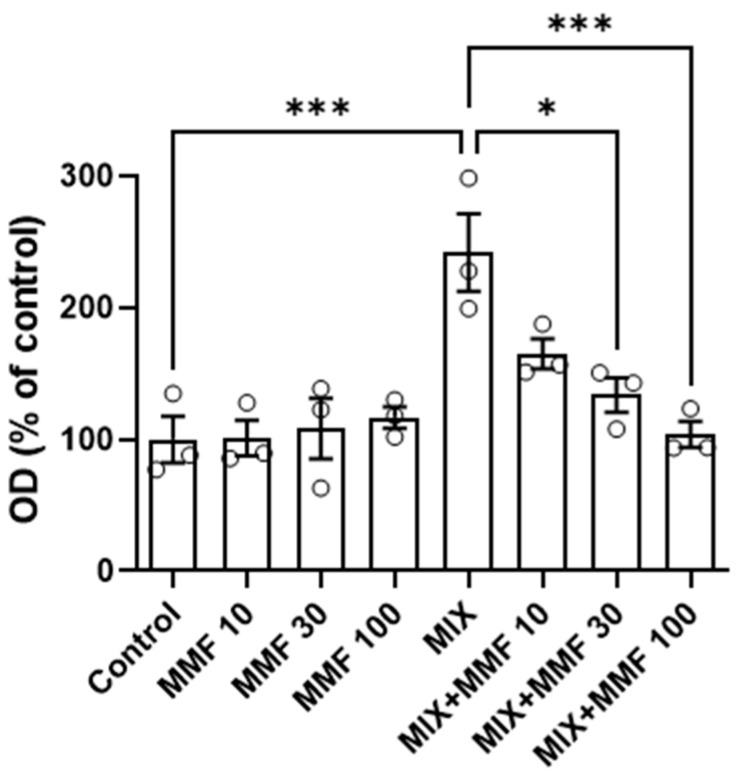
Effects of monomethyl fumarate (MMF) on differentiation mixture (MIX)-induced adipogenesis as assessed via the Oil Red O assay. The quantification of Oil Red O color is expressed as a percentage relative to the control condition. Data are expressed as the means ± standard deviation; * *p* < 0.05 and *** *p* < 0.001.

**Figure 4:**
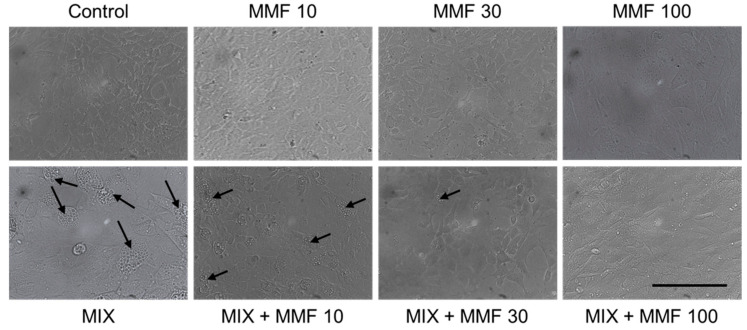
Visual confirmation of the effects of monomethyl fumarate (MMF) on adipogenesis. Phase-contrast microscopy images showing lipid droplet (arrows) formation in the differentiation mixture (MIX) groups in the absence and presence of the lowest concentration MMF; at a concentration of 100 µM, this phenomenon could not be observed, and morphological characteristics were like those of the control group. Magnification 2000× (objective 20×); scale bar = 200 µM.

The rate of differentiation of preadipocytes into adipocytes (adipogenesis) was measured via the Oil Red O assay. As shown in Figure 3, an increase in adipogenesis was observed in the MIX group compared to controls; an effect that was dose-dependently and significantly (for 30 and 100 µM) inhibited by MMF. MMF in the absence of MIX did not interfere with adipogenesis and was equal to control levels at all evaluated concentrations. The effects of MMF were further confirmed by phase-contrast microscopy (Figure 4). Formation of lipid droplets could clearly be observed in the MIX groups in the absence and presence of the lowest concentration of MMF. At a concentration of 100 µM MMF, droplet formation did not appear to occur, and morphological characteristics were like those of the control group, i.e., cells maintained their fibroblast-like phenotype.

### 3.3. MMF Prevents MIX-Induced (Adipogenesis-Associated) Expression of the Transcription Factors Peroxisome Proliferator-Activated Receptor Gamma (PPARγ) and CCAAT/Enhancer-Binding Protein Alpha (C/EBP-α)

**Figure 5:**
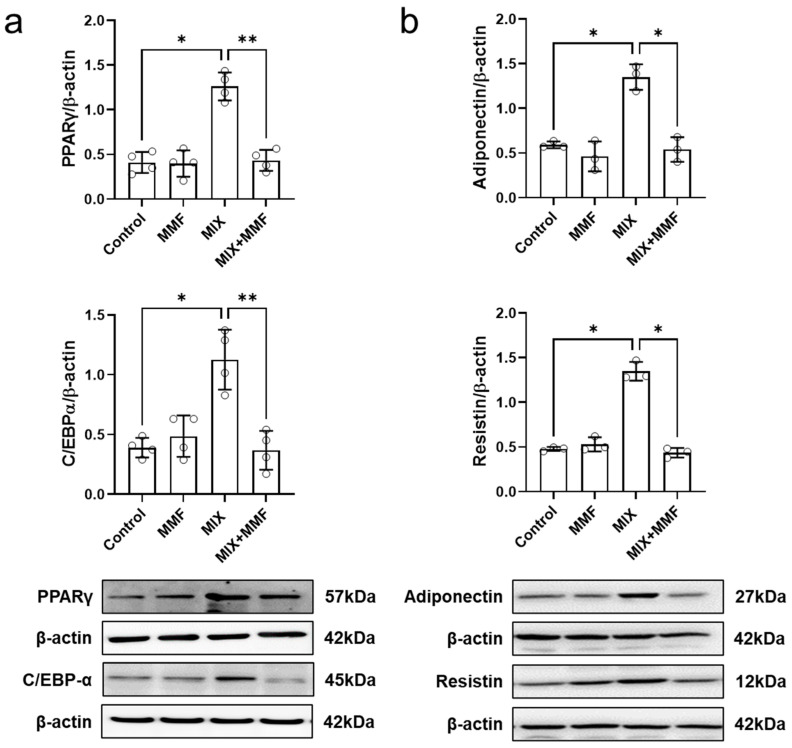
Monomethyl fumarate (MMF) fully suppresses differentiation mixture (MIX)-induced protein expression of the transcription factors peroxisome proliferator-activated receptor gamma (PPARγ) and CCAAT/enhancer-binding protein alpha (C/EBP-α) (**a**) as well as the adipokines, resistin and adiponectin (**b**). Cellular protein abundance was assessed by Western blotting. Data are expressed as the means ± standard deviation; * *p* < 0.05 and ** *p* < 0.01.

MIX markedly increased the protein expression of the transcription factors PPARγ and C/EBP-α, which could be fully prevented by co-incubation with 100 µM MMF (Figure 5a); these findings indicate MMF interferes with the expression of the key signaling molecules that drive adipogenesis. MMF alone did not affect the expression of these transcription factors.

### 3.4. MMF Suppresses MIX-Induced Adipokine Expression in Adipose Cells

Assessment of cellular adiponectin and resistin abundance via Western blotting revealed that MIX significantly induced the protein expression of these adipokines (Figure 5b). Importantly, these effects were fully prevented by MMF (100 µM) treatment, maintaining adipokine expression at control levels.

### 3.5. MMF Prevents High Triglyceride Levels Associated with Adipocyte Maturation

**Figure 6:**
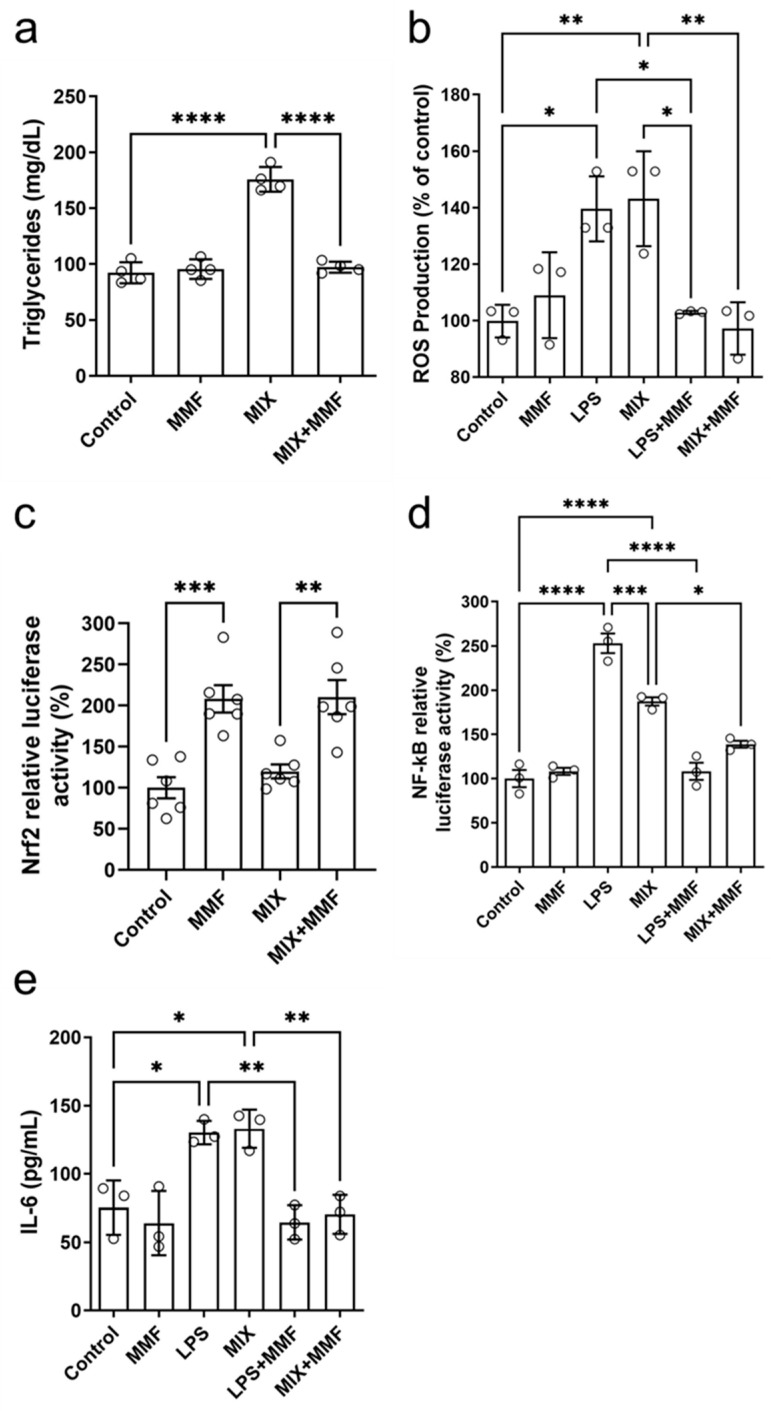
Effects of monomethyl fumarate (MMF) on various metabolic parameters. After seven days of differentiation, adipocyte triglyceride accumulation (**a**) was observed in the differentiation mixture (MIX) group; this was fully prevented in MIX cells co-incubated with MMF. In addition, MMF prevented lipopolysaccharide (LPS)- and MIX-induced increases in intracellular reactive oxygen species (ROS) (measured by the 2′,7′-Dichlorodihydrofluorescein diacetate [H2DCFDA] probe) (**b**). Panel (**c**) shows that MMF by itself induced gene transcription activity of the nuclear factor erythroid 2-related factor 2 (Nrf2) pathway (luciferase assay), a response unaffected by MIX. Furthermore, MMF inhibited the LPS- and MIX-induced increases in nuclear factor kappa B (NF-κB) activity (**d**) and IL-6 production (measured in supernatants) (**e**). Data are expressed as the means ± standard deviation; * *p* < 0.05, ** *p* < 0.01, *** *p* < 0.001 and **** *p* < 0.0001.

Mature adipocytes accumulate triglycerides intracellularly. Compared to the controls, MIX significantly increased the levels of these lipids; this effect was completely suppressed by MMF (Figure 6a). These findings further support that MMF prevents (MIX-induced) adipogenesis. No effects of MMF alone (in the absence of MIX) were observed.

### 3.6. MMF Prevents the Increase in Lipopolysaccharide (LPS)- and MIX-Induced Intracellular ROS Levels and Promotes Nrf2 Transcription

The treatment of 3T3-L1 cells with MIX stimulated the production of ROS to levels similar to those with LPS, which served as a positive control (Figure 6b). MMF alone did not affect ROS production but fully prevented the inductions by MIX and LPS. Figure 6c shows the results of Nrf2 gene transcription activity experiments by ARE activation through a specific luciferase assay. Interestingly, MMF by itself significantly activated the Nrf2 pathway, whereas MIX had no impact. Cells exposed to MIX+MMF showed similar Nrf2 activity as those incubated with just MMF, indicating MIX did not interfere with MMF effects on Nrf2 transcriptional activity.

### 3.7. MMF Prevents LPS- and MIX-Induced Increases in NF-κB Activity and IL-6 Levels

NF-κB activity was increased in the LPS (positive control) and MIX groups as compared to controls. These increases were (almost) completely prevented in the presence of MMF (Figure 6d). LPS and MIX stimulation of adipocytes significantly elevated supernatant levels of IL-6, which could be fully inhibited by MMF treatment (Figure 6e).

### 3.8. In Vivo Treatment with DMF Reduces HFHSD-Induced Weight Gain

**Figure 7:**
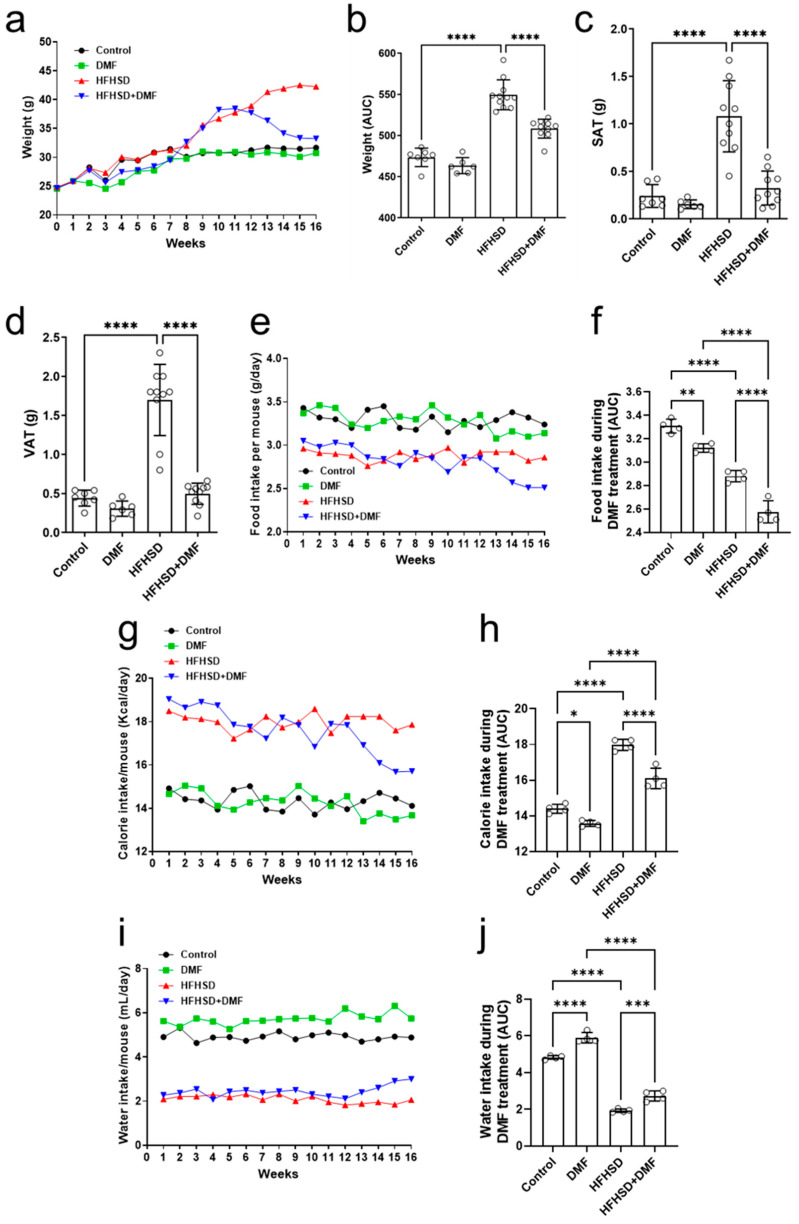
Dimethyl fumarate (DMF) reduces weight gain in high-fat/high-sucrose diet (HFHSD) mice. Animals were weighed for 16 consecutive weeks (**a**), and the area under the curve (AUC) was calculated during DMF treatment (**b**). Weight gain of HFHSD mice was greater than that of controls at the end of week 12, when treatment with DMF was started. Administration of DMF completely normalized body weight in HDHSD animals to control levels. DMF also strongly reduced HFHSD-induced increases in subcutaneous (SAT) (**c**) and visceral (VAT) (**d**) adipose tissue. Food (**e**,**f**), calorie (**g**,**h**), and water (**i**,**j**) intake were measured throughout the experimental period. Data are expressed as the means ± standard deviation; * *p* < 0.05, ** *p* < 0.01, *** *p* < 0.001 and **** *p* < 0.0001.

For the duration of the in vivo experiment, animals were weighed daily to evaluate body weight gain (Figure 7a). Mice in the control groups (standard diet ± DMF) experienced a baseline increase in body weight over the 16-week period, whereas animals that were fed HFHSD tended to exhibit additional weight gain, particularly in the final 8 weeks. Treatment with DMF was initiated at the start of week 13 and continued till the end of the experimental period. In HFHSD animals receiving DMF treatment, body weight gradually but considerably decreased over time (as compared to mice receiving HFHSD) to a level like that of animals receiving the standard diet. Evaluation of the area under the curve (AUC) (Figure 7b) confirmed the significant effects of DMF treatment on weight gain in the HFHSD group.

After euthanization of the animals at the end of week 16, the visceral and subcutaneous adipose tissues were weighed, and the tibias were measured. Figure 7c,d show increases in the mass of subcutaneous (SAT) and visceral adipose tissue (VAT), respectively, of HFHSD animals as compared to (standard diet) controls. Importantly, treatment with DMF normalized these increases to control levels. No differences in tibial length could be observed between experimental groups (Figure S5).

In addition to body weight and adipose tissue abundance, animal food intake was evaluated under the experimental conditions. Animals in the control groups (standard diet ± DMF) consumed constant amounts of food throughout the 16 weeks (Figure 7e). Food intake in the HFHSD group was also constant but lower than in the control and DMF-alone groups. Treatment with DMF of HFHSD mice resulted in a gradual food intake decline until the end of the experimental period. The AUCs indicated that DMF by itself reduced food intake to some extent as compared to the controls. HFHSD consistently reduced food intake, which was further enhanced by DMF treatment (Figure 7f). The average daily food intake per mouse, considering each individual weight within their respective groups, was also calculated. However, the results indicate that animals subjected to either a standard diet or the HFHSD exhibited a similar consumption pattern (Figure S6).

We also assessed the average caloric consumption in all experimental groups. Figure 7g shows that animals in the control group consumed constant amounts of calories throughout the 16 weeks, as did those in the DMF group, with a slight downward trend at the end of the experiment. Caloric consumption in HFHSD mice was constant as well but considerably higher than those in the standard diet groups. Treatment with DMF gradually decreased caloric intake in HFHSD animals. Evaluation of the AUCs confirmed a modest effect of DMF alone and its significant impact on calorie intake in HFHSD mice (Figure 7h).

Lastly, water consumption was significantly higher in the standard diet than the HFHSD groups (Figure 7i). However, treatment with DMF led to a substantial improvement in water intake in HFHSD animals (Figure 7j). The average daily water intake per mouse, considering each individual within their respective groups, was also calculated. However, the results indicate that animals subjected to either a standard diet or the HFHSD exhibited a similar consumption pattern (Figure S7).

### 3.9. DMF Prevents HFHSD-Induced Glucose Intolerance and Insulin Resistance

**Figure 8:**
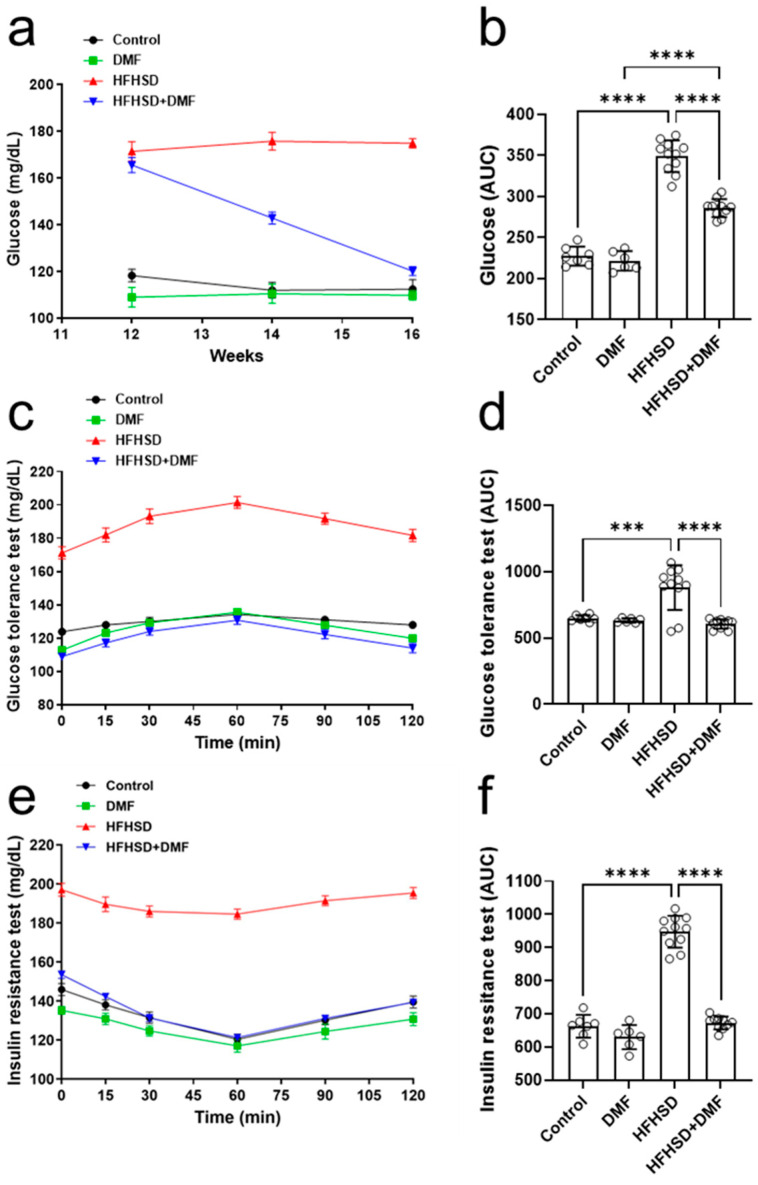
Dimethyl fumarate (DMF) treatment normalizes glucose metabolism in high-fat/high-sucrose diet (HFHSD) mice**.** Blood glucose levels were monitored throughout the experiment (**a**,**b**). Glucose tolerance (**c**,**d**) and insulin resistance (**e**,**f**) were tested as specified in the Materials and Methods section. Data are expressed as the means ± standard deviation; *** *p* < 0.001 and **** *p* < 0.0001.

The next step was to evaluate the fasting blood glucose levels of these animals. Figure 8a shows that the control and DMF-alone groups maintained stable blood glucose at 100–120 mg/dL, whereas the HFHSD group also maintained stable levels but within a much higher range of 160–180 mg/dL. Interestingly, the treatment of HFHSD animals with DMF strongly reduced the high fasting blood glucose levels after 2 weeks and normalized them to near control levels by the end of the experiment; these observations were further confirmed by analysis of the AUCs (Figure 8b).

Glucose tolerance (Figure 8c,d) was comparable between all groups, except for HFHSD, in which glucose levels were extremely high in blood samples; these findings suggest that DMF strongly improves glucose tolerance in HFHSD animals. The animals in the HFHSD group showed a significant degree of insulin resistance (Figure 8e,f).

Serum glucose levels were normalized to control levels in HFHSD animals treated with DMF, indicating DMF dramatically improves insulin sensitivity (Figure 8f).

### 3.10. DMF Modulates the Release of Inflammatory Cytokines in HFHSD Animals

**Figure 9:**
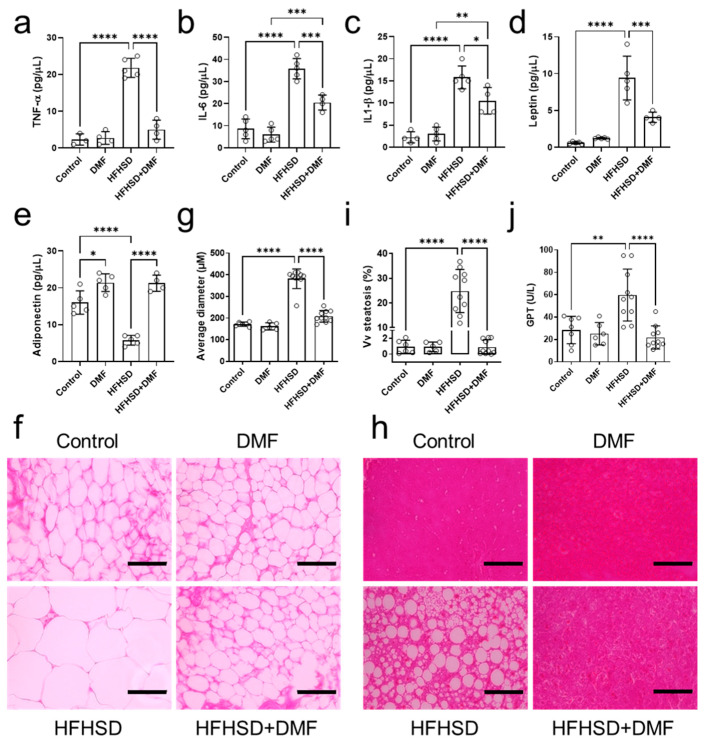
Dimethyl fumarate (DMF) treatment of high-fat/high-sucrose diet (HFHSD) mice regulates inflammatory mediators and improves histology. Plasma levels of tumor necrosis factor alpha (TNF-α) (**a**), interleukin (IL)-6 (**b**), IL-1β (**c**), leptin (**d**), and adiponectin (**e**) were determined in all groups. Panels (**f**,**g**) show representative histological images of visceral adipose tissue stained with hematoxylin and eosin (H&E) and the derived morphometric analysis, indicating HFHSD-induced adipocyte hypertrophy was prevented by DMF treatment. Histological analysis of the liver indicated accumulated lipid droplets in tissue from HFHSD animals, which is characteristic of hepatic steatosis (**h**). Further analysis confirmed that DMF prevented HFHSD-induced steatosis (**i**). Glutamate pyruvate transaminase (GPT) plasma levels indicated liver damage only in the HFHSD group (**j**). Together with the imaging results, this demonstrates DMF administration has no hepatotoxic effects. Data are expressed as the means ± standard deviation; * *p* < 0.05, ** *p* < 0.01, *** *p* < 0.001 and **** *p* < 0.0001.

Levels of TNF-α, IL-6, and IL-1β were elevated in the plasma of HFHSD animals compared to controls (Figure 9a–c). These increases were fully (TNF-α) or partially (IL-6 and IL-1β) reduced by treatment with DMF.

As compared to the control group, adiponectin levels were lower in the HFHSD and higher in the DMF and HFHSD + DMF groups (Figure 9e). These observations demonstrate that HFHSD decreases adiponectin and suggest that DMF can increase adiponectin levels regardless of the diet. Leptin concentrations were elevated in the HFHSD group, indicative of leptin resistance; levels were reduced by DMF treatment but remained significantly higher than those in control plasma (Figure 9d).

### 3.11. DMF Prevents Visceral Adipose Tissue Hypertrophy and Liver Tissue Steatosis in HFHSD Mice

Visceral adipose tissue was collected to prepare histological slides for the evaluation of morphological characteristics (hyperplasia or hypertrophy) by quantifying the average diameter of adipocytes stained with H&E. The standard diet groups presented with characteristic adipocytes. In contrast, HFHSD animals exhibited hypertrophic adipocytes. Importantly, HFHSD animals treated with DMF showed adipocytes with morphological characteristics like those of the control group (Figure 9f,h). Of note, all images presented were acquired at the same magnification.

Histological slides were also prepared from liver tissues to evaluate ectopic fat deposition (hepatic steatosis) and potential DMF-induced liver toxicity. Figure 9h shows representative histological images of liver tissue from the experimental groups; no morphological variations to indicate DMF toxicity could be detected. However, we did observe lipid droplet formation (steatosis) in the HFHSD group, which was measured and is presented in Figure 9i. As indicated by imaging and confirmed by quantification, DMF treatment fully prevented steatosis development in HFHSD animals. Lastly, GPT plasma levels, as an indicator of liver injury, were elevated in the HFHSD group, which could be fully suppressed in animals treated with DMF; collectively, these findings indicate no hepatotoxicity because of DMF administration.

## 4. Discussion

This study aimed to assess the effects of fumaric ester treatment (DMF in vivo and MMF in vitro) on metabolic disorders related to obesity, and identify the key molecular mechanisms involved. Initial experiments demonstrated that MMF (i.e., the main active metabolite of DMF that reaches the systemic circulation and exerts therapeutic effects) at concentrations of 10, 30, and 100 µM had no adverse effects on cell viability. Preliminary results indicated 100 µM was most effective in terms of cellular responses and was selected for subsequent investigations. Our in vitro studies revealed that exposure to MMF prevents the development of adipogenesis by reducing the expression of transcription factors that drive the differentiation of preadipocytes into mature adipocytes and by decreasing triglyceride levels. In addition, we observed antioxidant and anti-inflammatory effects as evidenced by reductions in ROS, adipokine, proinflammatory cytokine IL-6 and resistin levels after treatment with MMF. The antioxidant and inflammatory actions were further confirmed by MMF-mediated stimulation and inhibition of Nrf2 and NF-κB transcription factor activities, respectively. In vivo, the choice to use DMF instead of MMF was supported by significant technical and clinical considerations [19]. DMF has already been established as a pharmacological agent with clinical applications in the treatment of various conditions; considering DMF is already an approved clinically recognized medicine and the availability of extensive information on its pharmacokinetics, toxicity, and safety profile validate the use of this fumaric ester in our in vivo protocol [21,28,29].

Here, in vivo tests were conducted in mice subjected to a high-fat/high-sucrose diet (HFHSD) and treated with DMF, mimicking the clinical approach for the treatment of obese patients [16]. DMF was found to reduce fat mass and food consumption, suggesting it plays a role in modulating leptin, a hormone responsible for controlling food intake. Indeed, leptin levels were strongly reduced in HFHSD animals receiving treatment, suggesting that DMF exerts an anorectic effect. HFHSD may initially increase calorie intake due to its palatability, but long-term exposure can lead to appetite suppression through a combination of hormonal, neural, and gut microbiota changes. While we cannot rule out other causes, we suggest that the reduction in HFHSD consumption in our study may be linked to the observed changes in leptin levels. The glycemic profiles of HFHSD mice showed an almost complete normalization to control levels in fasting blood glucose, insulin response, and glucose tolerance after treatment with DMF. In addition, a considerable reduction in obesity-associated low-grade chronic inflammation (TNF-α, IL-6, IL-1β plasma levels) could be observed. DMF was also able to prevent or reverse the development of cellular hypertrophy in adipose tissues and the ectopic deposition of lipids in the liver, a typical feature of hepatic steatosis concomitant with a reduction in GPT levels, a marker of liver injury. DMF has emerged as a highly relevant candidate for the treatment of obesity in line with our findings; previous work showed equivalent results of DMF on adipocyte hypertrophy and steatosis [26].

Following maturation, adipocytes begin to retain lipids, particularly triglycerides [7,30,31,32]. The MIX group showed a significant increase in triglyceride accumulation, indicating the complete differentiation of preadipocytes into mature adipocytes. As indicated, co-incubation with MMF prevented this deposition by interfering with the differentiation process; the observed reduction in triglyceride levels was a direct consequence of fewer mature adipocytes [33]. Transcription factors such as PPARγ and C/EBP-α play crucial roles in regulating adipocyte differentiation [1,34,35,36,37]. In this study, MMF inhibited MIX-induced protein expression of these transcription proteins, thereby interfering with preadipocyte differentiation. Accordingly, the histological analysis of the visceral adipose tissue revealed that oral treatment of HFHSD mice with DMF fully prevented the accumulation of hypertrophic adipocytes. Both transcription factors and ROS are important regulators of adipogenesis. A recent study showed that peroxiredoxin 2 (Prx2), a transmembrane ROS scavenger protein, is increased during adipogenesis, which induces the differentiation of 3T3-L1 cells into adipocytes [38]. This process is inhibited not by a reduction in transcription factors PPARγ and C/EBP-α but by the accumulation of intracellular ROS, leading to cell death [39]. We demonstrated that MMF was able to activate the Nrf2/ARE pathway, which underpins its inhibiting effects on MIX-induced total ROS production in vitro during adipogenesis. These results indicate that adipogenesis requires an increase in ROS production, which involves insufficient antioxidant pathway activity. Prx2 is activated by DMF, controlling ROS production via antioxidant transcription [38]; involvement of this scavenger protein in the observed effects requires future investigations.

Adiponectin, a hormone crucial for carbohydrate and lipid metabolism in insulin-sensitive tissues, plays a significant role in insulin sensitization [40]. It promotes the oxidation of fatty acids, regulates glucose uptake in muscles, and inhibits glucose synthesis in the liver [11]. The release of adiponectin is inhibited by proinflammatory cytokines, suggesting inflammation may contribute to hypoadiponectinemia in the context of insulin resistance, a phenomenon often occurring in obese individuals [41]. In vitro, the decrease in adiponectin levels in the MMF-treated group may be explained by MMF’s inhibition of preadipocyte differentiation, which prevents the formation of fully mature adipocytes responsible for adiponectin secretion. Conversely, in the in vivo model, mice fed an HFHSD showed reduced adiponectin expression, consistent with adipose tissue dysfunction, leading to a pro-inflammatory state (TNF-α, IL-6, IL-1β) and decreased adiponectin production. However, in mice treated with DMF, an increase in adiponectin levels was observed, suggesting that DMF improved adipose tissue function, potentially by reducing obesity-associated inflammation and restoring adiponectin secretion. These results highlight the fundamental differences between the two experimental models: while MMF inhibits cell differentiation in the in vitro model, resulting in lower adiponectin secretion due to the absence of mature adipocytes, DMF exerts an anti-inflammatory effect on adipose tissue in the in vivo model, increasing adiponectin production in already differentiated adipocytes. In both cases, the compounds modulate adipogenesis and adipose tissue function, although through distinct mechanisms that directly influence adiponectin levels. Although no conclusive scientific evidence is available to elaborate on the mechanisms driving this regulation by DMF, studies indicate the involvement of the redox-sensitive NF-κB pathway activated during oxidative stress [42,43]. This could explain the reduction in proinflammatory mediators and the attenuation of low-grade chronic inflammation by DMF treatment in obese animals. The link between adiponectin deficiency and the development of insulin resistance, obesity, and type 2 diabetes is well documented [12,44,45,46]. Plasma analyses revealed that untreated HFHSD mice exhibited high fasting glycemia. These animals demonstrated glucose intolerance and insulin resistance, aligning with the effects of adiponectin deficiency and unfavorable metabolic states observed in individuals with type 2 diabetes [47,48,49,50]. The interrelationship between adiponectin levels, inflammation, insulin resistance, and the effects of DMF highlights the complexity of the mechanisms underlying these metabolic responses. Insulin resistance has been associated with elevated levels of resistin [32,51]. Interestingly, resistin has been linked to increased ROS generation and oxidative stress [11,26,32,52]. The findings of this study that show abundant resistin expression in mature adipocytes corroborate and complement existing knowledge about the mechanisms involved in insulin resistance during obesity, indicating the strong involvement of oxidative stress.

DMF treatment led to a significant reduction in body mass in obese mice, whereas untreated HFHSD animals continued to gain weight. Interestingly, animals on an HFHSD consistently consumed smaller amounts of feed than those provided with a standard diet, suggesting caloric intake may be the determining factor for the development of obesity. As expected, caloric intake was significantly greater in HFHSD mice and could be markedly reduced by DMF treatment. These findings suggest possible central-level regulation by DMF. Leptin plays a crucial role in satiety regulation; elevated levels of circulating leptin are common in obese individuals despite their resistance to leptin [53,54,55]. Our results indicate that DMF treatment significantly suppresses plasma levels in HFHSD animals. This apparent relationship between leptin (resistance) and DMF effects illustrates the intricate interplay between endocrine processes, treatment response, and metabolic physiology.

Hepatic steatosis, a process characterized by lipid accumulation in hepatocytes, is a condition that can lead to liver scarring and cirrhosis [44,56,57]. In NAFLD/NASH patients, there is an imbalance between the synthesis and export of triglycerides, leading to their accumulation in hepatocytes [44,58]. Hepatocytes can take up glucose from the blood when other cells become resistant to insulin, resulting in increased production of glucose and triglycerides, which contributes to fat accumulation in the organ [54,59]. GPT is an enzyme found primarily in the liver and often measured as a marker of liver function, as its levels rise when liver cells are damaged, for example during NAFLD/NASH. A remarkable finding of this study was that DMF can reverse this condition, even after the onset of hepatic steatosis.

Several studies have explored the use of DMF and MMF, primarily known for their immunomodulatory and anti-inflammatory properties, in the treatment of multiple sclerosis and psoriasis [21,28,60,61,62]. However, their potential roles in metabolic regulation are being timidly studied. In a stroke recovery model [63], DMF and MMF were found to influence recovery in mice through their action on the Nrf2 pathway, which is linked to oxidative stress reduction and inflammation modulation. Although this study was focused on cerebral ischemia, it suggests that both DMF and MMF can mitigate cellular damage through these pathways; a mechanism that may extend to other tissues such as adipose tissue. Most relevant research currently available focuses on MMF and DMF’s pharmacokinetics and their effects in neurological and immune contexts, suggesting the need for future studies to examine their role in metabolism and adipogenesis.

This is the second study from our group demonstrating anti-obesogenic effects, and the first to suggest that DMF can alter the course of obesity. Despite these novel findings, we acknowledge several limitations in our study. The in vitro study, given the experimental limitations, was performed with only three discrete MMF concentrations, rather than as a complete dose-response curve. The in vivo study was conducted in mice and with one single dose of DMF, and drug metabolism differs significantly between mice and humans. Therefore, the findings on obesity control by DMF may not directly translate to obese patients. We were unable to assess the histology and function of other metabolically relevant organs, such as the heart, kidneys, and intestines. Although our results suggest that HFHSD and DMF are not toxic in the form and dose provided to the mice, we cannot completely rule out this possibility. Additionally, the dose of DMF used in our study corresponds allometrically to two to four times the dose typically used in patients. Future studies will need to examine the dose-response relationship, as we have done in vitro. Moreover, we do not have direct evidence that DMF increased Nrf2 expression or decreased NF-kB expression in vivo, despite suggestive outcomes in the liver and adipose tissue histology, cytokine and adipokine profiles, and glucose metabolism. It is unclear how the effects on Nrf2 would lead to appetite suppression. Leptin levels is only a clue, but this warrants more detailed investigation. Key hepatic antioxidant markers, such as reduced glutathione, oxidized glutathione, and glutathione transferase, were not analyzed, which could potentially indicate a Michael reaction between DMF and thiols. In addition, we were unable to measure the markers of oxidative stress in this study. Since DMF is an antioxidant and it would be straightforward to suggest its role in regulating oxidative stress, we chose to focus on analyzing inflammatory parameters, which are more commonly studied in patients undergoing DMF treatment. Lastly, although we used the well-established C57BL6 mouse model for HFHSD-induced obesity, we were unable to replicate this experimental design in transgenic and/or Nrf2 knockout mice of the same strain background.

## 5. Conclusions

In summary, this study underscores the therapeutic potential of fumaric esters such as DMF for the treatment of obesity and related metabolic disorders by interfering with adipogenesis through the mechanisms impacting transcription factor activation, proinflammatory cytokine release, and antioxidant pathway activation.

## Figures and Tables

**Table 1:** List of antibodies and dilutions.

Antibody	Dilution	Manufacturer	Source
Adiponectin	1:1000	Boster	Rabbit polyclonal
β-actin	1:1000	Sigma-Merck	Mouse monoclonal
C/EBP-α	1:1000	Invitrogen	Rabbit polyclonal
PPARγ	1:1000	Invitrogen	Rabbit polyclonal
Resistin	1:1000	Boster	Rabbit polyclonal
Anti-mouse	1:5000	Sigma-Merck	Donkey
Anti-rabbit	1:5000	Invitrogen	Goat

Antibodies used for Western blotting in cell homogenates.

## Data Availability

The data from this study will be available to interested parties upon request.

## Supplementary Materials

The following supporting information can be downloaded at https://www.mdpi.com/article/10.3390/antiox13121496/s1, detailed diet, original blotting membranes (uncropped), MTT additional tests and left tibia measurements.
