# Supplementary material for: Therapeutic Potential of Dimethyl Fumarate for the Treatment of High-Fat/High-Sucrose Diet-Induced Obesity
Source: Antioxidants (Basel). 2024 Dec 8;13(12):1496. doi: 10.3390/antiox13121496 (PMC11673011; doi:10.3390/antiox13121496)
Supplement: Supplementary file 1 [file antioxidants-13-01496-s001.zip › antioxidants-3366069-supplementary.pdf]

Supplementary material

Dimethyl fumarate regulates high-fat and high-sucrose diet-induced obesity

Helber da Maia Valenca[a], Evelyn Caribé Mota[a], Andressa Caetano da Fonseca Andrade Silva[a], Alexsandro Tavares Figueiredo-Junior[a], Fernanda Verdini[a], Bruna Romana-Souza[b], Mariana Renovato-Martins[c], Manuella Lanzetti[a], Samuel dos Santos Valenca*[a], João Alfredo de Moraes[a]

* corresponding author

a) Federal University of Rio de Janeiro (UFRJ). Institute of Biomedical Sciences, Avenida Carlos Chagas Filho 373, bloco F, 3° floor, room 301. Cidade Universitária, Rio de Janeiro, RJ, ZIP 21 941-902, Brazil;
b) State University of Rio de Janeiro (UERJ), Department of Histology and Embryology, Rua Professor Manoel de Abreu, 444, 3° andar, Rio de Janeiro, RJ, ZIP 20550-170, Brazil;
c) Fluminense Federal University (UFF), Departamento de Biologia Celular e Molecular, Instituto de Biologia, Laboratório de Inflamação e Metabolismo, Rua Professor Marcos Waldemar de Freitas Reis, s/n, 24020-140, Niterói, RJ, ZIP 20550-170, Brazil.

Diet

The high-fat and high-sucrose diet (HFHSD) and the standard diet were prepared by the company PRAGSOLUÇÕES® (PRAGSOLUÇÕES, São Paulo, Brazil) in accordance with the recommendations of the American Institute of Nutrition (AIN-93 M). For detailed information about the diet, see the supplemental. Both diets were offered to the animals (according to the group) ad libitum. Detailed information about the standard diet is found below:

MAIZE STARCH	46.570%
CASEIN	14,000%
DEXTRINIZED STARCH	15.500%
SUCROSE	10,000%
SOY OIL	4,000%
MICROCRYSTALLINE CELLULOSE	5,000%
MINERAL MIX AIN 93 M	3,500%
MIX VIT AIN 93	1,000%
L CYSTINE	0.180%
CHOLINE BITARTART	0.250%
B HT	0.001%
	100.001%

Figure S1. Detailed information about standard diet

MAIZE STARCH	9.270%
CASEIN	14,000%
SUCROSE	40.280%
SOY OIL	3.630%
MICROCRYSTALLINE CELLULOSE	5.820%
MINERAL MIX PSB10026	1,160%
MIX VIT AIN 93	1,160%
L CYSTINE	0.180%
CHOLINE BITARTART	0.260%
DIBASE CALCIUM PHOSPHATE	1,680%
CALCIUM CARBONATE	0.640%
POTASSIUM CITRATE	1,920%
LARD	20.0000%
	100.000%

Figure S2. Detailed information about the HFHSD

Electrophoresis and Western blotting

The membranes were incubated at with primary antibodies overnight. Uncropped and unedited membranes images are below:

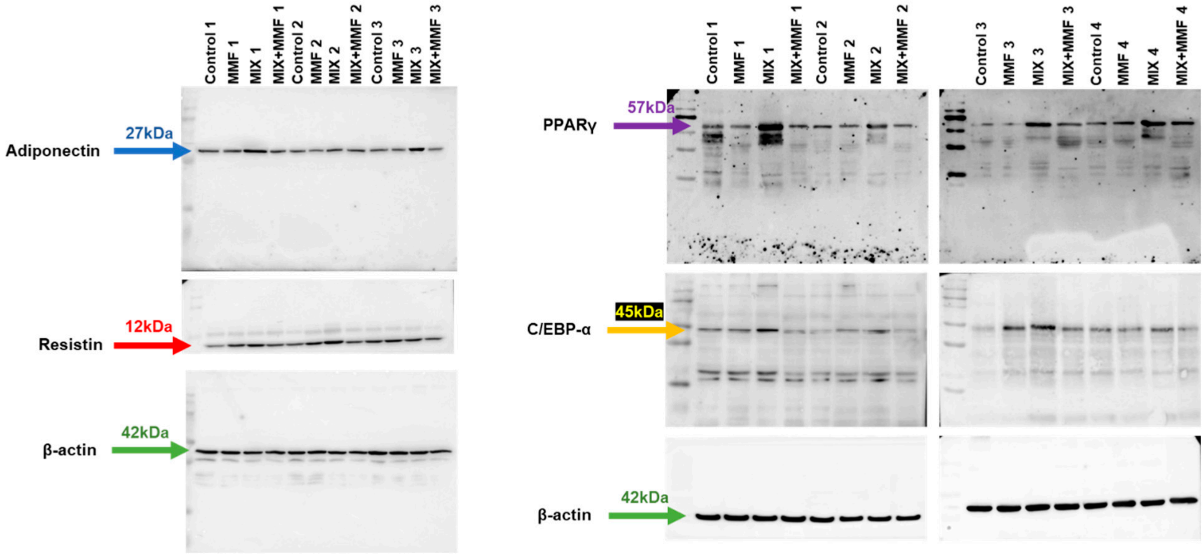

Figure S3. Uncropped membranes

MMF did not affect metabolic activity at various concentrations or for various durations

According to the results of the MTT assay, MMF did not affect the viability of 3T3-L1 cells at concentrations of 10, 30, and 100 μM after 24, 48, and 96 hours (Figure 1, 2 and 3, respectively). The results were statistically similar to those of the control group. DMSO was used as the solvent for diluting MMF, and it did not impact the metabolic viability of the cells at any of the concentrations tested. There were no significant differences observed between the groups. Compared with the control group (DMEM 1%), the DMEM 10% calf serum group (positive control) presented higher proliferation rates.

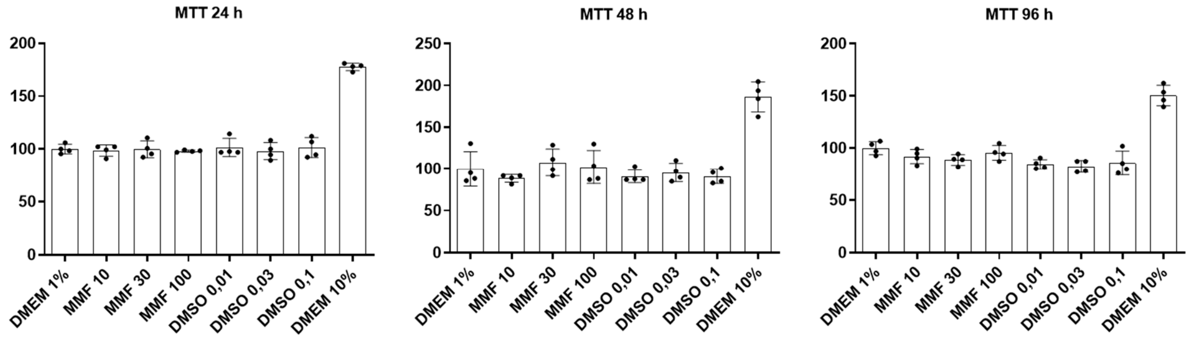

Figure S4. MTT from different concentrations of MMF or DMSO

Left tibia measurement

Measurement of left tibial length showed that none of the experimental groups had a significant difference (see below).

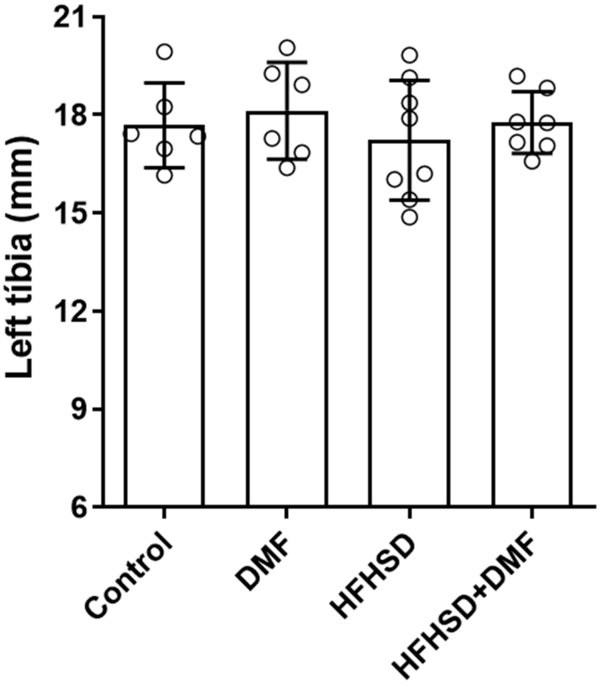

Figure S5. Left tibia measurement was similar among the groups.

Additional calculation for food and water intake

Water and food intakes were normalized per g of body weight per day since mice differs among the groups, so this can also produce some differences in water and food intake.

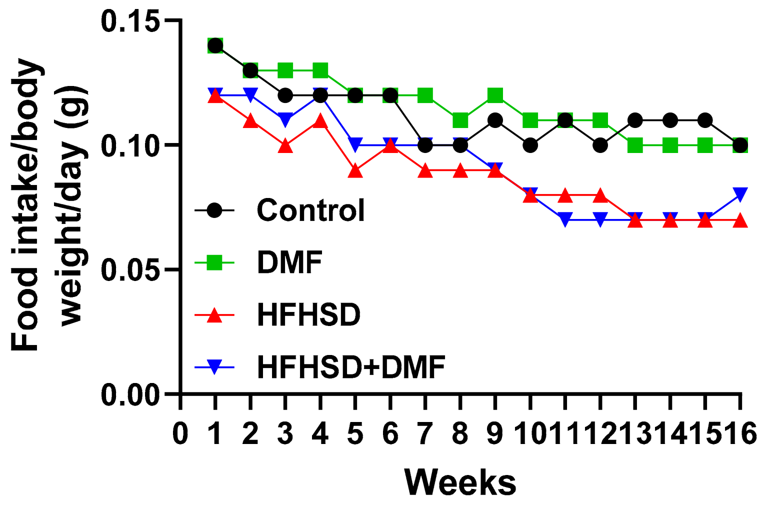

Figure S6. The average daily food intake per mouse, considering each individual within their respective groups. Results indicate that animals subjected to either a standard diet or the HFHSD exhibit a similar consumption pattern.

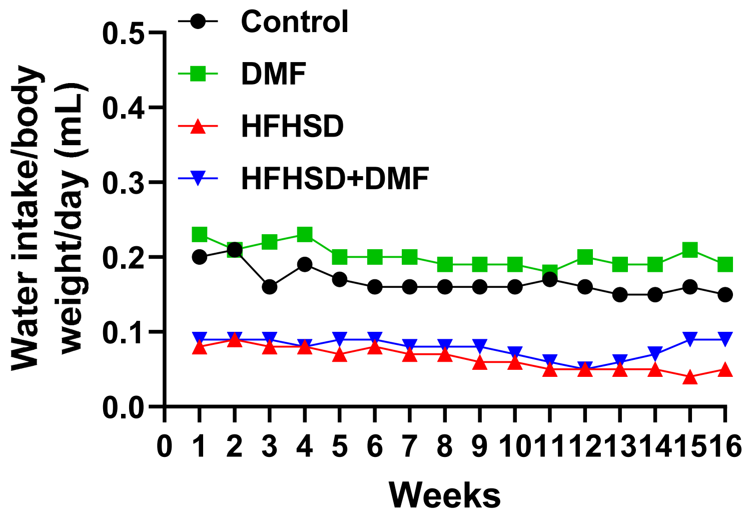

Figure S7. The average daily water intake per mouse, considering each individual within their respective groups. Results indicate that animals subjected to either a standard diet or the HFHSD exhibit a similar consumption pattern.
